# Effectiveness of a person-centred eHealth intervention in reducing symptoms of burnout in patients with common mental disorders – secondary outcome analysis of a randomized controlled trial

**DOI:** 10.1186/s12875-023-02172-9

**Published:** 2023-10-19

**Authors:** Sara Alsén, Emina Hadžibajramović, Ingibjörg H Jonsdottir, Lilas Ali, Andreas Fors

**Affiliations:** 1 Institute of Health and Care Sciences, Sahlgrenska Academy, University of Gothenburg, Box 457, Gothenburg, 405 30 Sweden; 2 Gothenburg Centre for Person-Centred Care (GPCC), University of Gothenburg, Gothenburg, Sweden; 3 Institute of Stress Medicine, Region Västra Götaland, Gothenburg, Sweden; 4 School of Public Health and Community Medicine, Sahlgrenska Academy, University of Gothenburg, Gothenburg, Sweden; 5 Psychiatric Department, Sahlgrenska University Hospital, Gothenburg, Sweden; 6 Research, Education, Development and Innovation, Primary Health Care, Region Västra Götaland, Gothenburg, Sweden

**Keywords:** Depression, Anxiety, Stress, Exhaustion disorder, Shirom-Melamed Burnout Questionnaire, Patient-centred care, Person-centred care, Telehealth, mHealth, Intervention, Randomized controlled trial

## Abstract

**Background:**

The number of people with common mental disorders (CMDs), especially stress-related disorders, has increased in several countries, including Sweden, during the past decade. Patients seeking care for long-term stress report severe symptoms. Although person-centred care (PCC) has shown several benefits, studies evaluating the effects of a PCC eHealth intervention on patients with CMDs are scarce.

**Objective:**

The aim of this study was to compare levels of self-reported symptoms of burnout between a control group receiving treatment as usual (TAU) and an intervention group receiving TAU with the addition of a person-centred eHealth intervention, in patients on sick leave for CMDs.

**Methods:**

This study reports analysis of a secondary outcome measure from a randomized controlled trial. Patients (n = 209) on sick leave for CMDs were recruited from nine primary health care centres and allocated to either a control group (n = 107) or an intervention group (n = 102). The intervention consisted of phone support and an interactive digital platform built on PCC principles. Self-reported symptoms of burnout were assessed using the Shirom-Melamed Burnout Questionnaire (SMBQ) at baseline and at 3 and 6 months.

**Results:**

Our findings showed changes in SMBQ scores over time in both the control and the intervention group. There was no significant difference in SMBQ scores between the groups; however, a difference in change over time between the groups was observed. The SMBQ scores decreased significantly more in the intervention group than in the controls between 0 and 3 months and between 0 and 6 months. No differences in change between the two groups were seen between the 3- and 6-month follow-ups.

**Conclusion:**

This person-centred eHealth intervention for patients on sick leave for CMDs showed a slight initial effect in reducing symptoms of burnout. Taking into account that both groups reported comparable SMBQ scores throughout the study period, the overall effect may be considered limited.

**Trial registration:**

: The trial was registered in ClinicalTrials.gov (Identifier NCT03404583). Date of registration: 19/01/2018. https://clinicaltrials.gov/ct2/show/NCT03404583.

## Introduction

Internationally, the term “common mental disorders (CMDs)” often refers to various anxiety and depression disorders [1]. However, the conditions included in the term may vary and have, in several studies, been expanded to also include stress-related disorders, e.g., adjustment disorder, reaction to acute stress, burnout, or exhaustion [2–4]. Mental disorders are a significant issue for many countries and health care systems. They are related to considerable losses in health and functioning, causing high sick leave spells with a long mean duration and early retirement [5–7]. In Sweden, the number of people with CMDs, especially stress-related disorders, has increased during the past decade [8] and accounts for an increasing proportion of sick leave [9]. Therefore, stress-related disorders need to be assessed and detected early in the healthcare process to reduce the risk of the increased severity of the condition.

Depression and anxiety are the most common mental health diagnoses among patients in primary care and symptoms of depression and anxiety are strongly related to psychosocial stress [10]. Many patients also seek care for symptoms of severe mental and physical exhaustion and cognitive impairment clearly related to long-term stress. These symptoms are considered core components of burnout [11–13], but which diagnosis is set for these patients varies greatly. Indeed, the term “clinical burnout”, usually based on the criteria of work-related neurasthenia in the International Classification of Diseases, ICD-10, is used in several countries. Still, a complication for clinicians is that the “burnout” definition is heterogeneous, and no homogenous diagnostic criteria have been established. Attempts have been made to adapt the burnout concept to be more usable in clinical practice, but it has been found that the most utilized burnout tool, the Maslach Burnout Inventory, does not seem suitable as a diagnostic tool for patients [14]. In the International Classification of Diseases and Related Health Problems (ICD), burnout is classified under “Problems related to life management difficulty” (Z73), but even here, it is not considered a disorder [15].

In Sweden, the diagnosis “exhaustion disorder (ED)”, has been introduced in the Swedish version of the ICD, 10th revision (ICD-10) with diagnostic code F43.8 A. The diagnostic criteria state that at least one identified stressor, work- or non–work-related, should have been present for at least six months and that the clinical picture is dominated by a lack of psychological energy. Four of the following symptoms should have been present almost every day for at least two weeks: concentration or memory impairment, emotional instability, reduced ability to cope with demands and/or time pressure, disturbed sleep, apparent physical weakness, and physical symptoms such as muscular pain [16, 17]. Exhaustion disorder is applicable in clinical practice and corresponds with the concept of clinical burnout [16]. This criteria-based diagnosis describes patients seeking care for symptoms of exhaustion due to work-related or non-work-related stress exposure, lasting at least 6 months [17]. Regardless of diagnosis, severity of illness related to stress is warranted to measure in patients with mental health problems and a questionnaire often used and well suited to measure the severity of illness and treatment outcomes both in a working population and in a clinical setting is the Shirom-Melamed Burnout Questionnaire (SMBQ) [18–20].

Among a population of working-age patients seeking primary care, regardless of the reason, 59% reported stress-related symptoms, with high symptoms of burnout and exhaustion [21]. Patients with ED commonly report a high burden of mental symptoms, and comorbidity with depression and anxiety is frequent [22]. Many patients also report long-lasting symptoms and reduced work ability, and as many as 63% report that they made some changes at work years after the onset of exhaustion [9, 23, 24]. The patients often seek care for anxiety, depression and stress-related complaints years preceding the ED [25]. A longer symptom duration before receiving a stress-related diagnosis is associated with a prolonged rehabilitation process [26], indicating that primary care is essential for early detection and support. In Sweden, the national guidelines recommend that treatment for depression and anxiety disorders consist of medication, cognitive behavioural therapy, or both [27]. For stress-related disorders, no recommended national guidelines have been published [28]. Most consultations and treatments regarding CMDs occur in primary care [29], where it is a challenge to meet the needs of these patients. eHealth interventions have shown to be a feasible option for face-to-face treatments [30, 31], facilitating self-management, with increased accessibility and direct involvement of the patient [32]. The need to provide support also at a distance has been especially emphasized during the COVID-19 pandemic [33].

Person-centred care (PCC) aims to engage the patient as an active partner in their care and treatment. It is based on ethical principles emphasizing the importance of knowing the patient as a person with resources and needs, which is essential for establishing a mutually respectful partnership between the patient (often including relatives) and health care professionals (HCPs) [34, 35]. Studies evaluating interventions based on PCC (provided face to face or remotely) in various health care settings, targeting multiple conditions, have shown positive effects, such as increased self-efficacy, shortened length of hospital stay, improved satisfaction with care, reduced symptom burden, and cost savings [36]. However, as far as we know, no studies have investigated whether a PCC eHealth intervention affects self-reported symptoms of burnout in patients on sick leave for CMDs.

This study aimed to compare levels of self-reported symptoms of burnout between a control group receiving treatment as usual (TAU), and an intervention group receiving TAU with the addition of a person-centred eHealth intervention, in patients on sick leave for CMDs. Three research questions were formulated: Does the SMBQ score change over time? Does the SMBQ score differ between the groups over time? Does the change in SMBQ score differ between the groups over time?

## Methods

### Study design

This article reports a secondary outcome analysis of the PROMISE study (“Person-centred eHealth for treatment and rehabilitation of common mental disorders”), which was an open randomized controlled trial (RCT) evaluating the effects of a six-month person-centred eHealth intervention (interactive digital platform and telephone support) for patients on sick leave for CMDs. Study methods and procedures of the PROMISE study have been presented elsewhere [37] but are briefly described below.

### Setting and participants

**Fig. 1:**
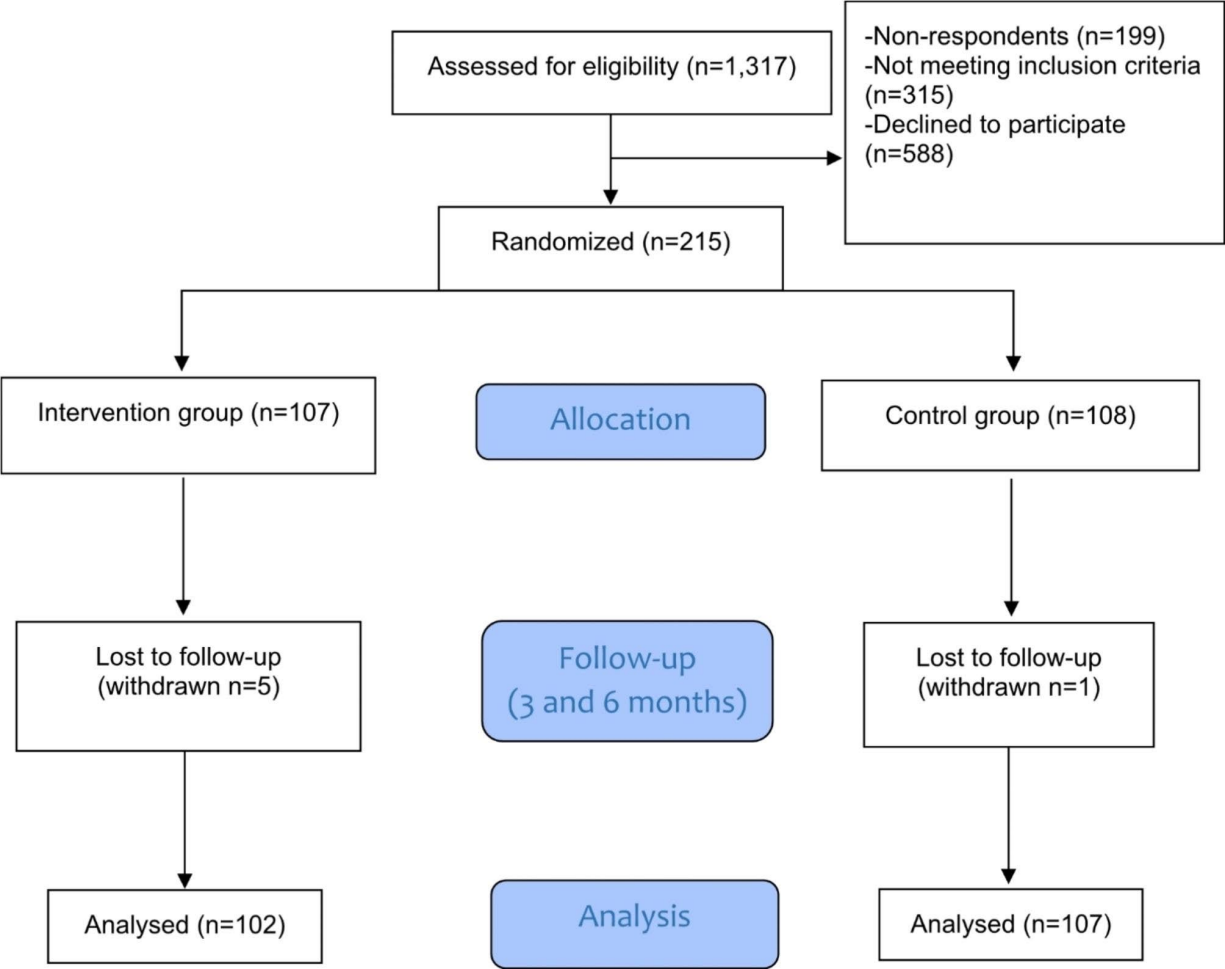
CONSORT diagram

Eligible patients at nine public primary health care centres in western Sweden were screened by designated HCPs and enrolled between February 2018 and June 2020. Patients were considered eligible if they were 18–65 years old, and currently employed or studying (at least part-time) during the last 9 months, had a registered address, could understand written and spoken Swedish, were currently on sick leave that had lasted no longer than 30 days, and had been diagnosed by a physician for any of the following conditions in ICD-10: mild to moderate depression (F32 and F33), mild to moderate anxiety disorder (F41), reaction to severe stress, and adjustment disorders (F43, which includes the Swedish diagnosis ED (F43.8 A)). Exclusion criteria were previous sick leave exceeding 14 days for any of the diagnoses mentioned above during the last 3 months, severe impairment hindering the use of the eHealth intervention, ongoing documented alcohol or drug abuse, severe disease that had a survival expectancy of < 1 year or that could interfere with follow-up if the intervention was assessed as a burden, or participation in a conflicting study. A flowchart of the study participants is presented in Fig. 1.

Eligible patients were sent an information letter about the study by regular mail and informed that an HCP would be in contact with further details and to ask if they would like to participate in the study. They were also informed that they could contact the HCPs and/or the researchers for more information about the study. Patients who agreed to participate were sent a consent form to sign and return in a prepaid envelope. Randomization was based on a computer-generated list created by a third party and stratified by age (< 50 or ≥ 50 years) and diagnostic group (1: Depression, 2: Anxiety, 3: Stress reactions and disorders). Ethical approval was obtained from the Regional Ethical Review Board in Gothenburg, Sweden (DNr 497 − 17, T023-18, and T526-18).

In total, 1,317 patients were screened for study enrolment. Of the 1,118 patients who were reached by phone, 803 met the eligibility criteria, 588 of whom declined to participate. The remaining 215 patients were randomized and assigned to either the control or the intervention group. Six of these withdrew consent, leaving 107 in the control group and 102 in the intervention group. All participants were informed of their allocation by phone.

### Control group

Patients enrolled to the control group received TAU, which for patients with CMDs often takes place in primary care. Initially, they consult a physician to initiate the consultation, treatment, and follow-up on sick leave. Treatment often consists of medication or cognitive behaviour therapy, or both [27]. However, treatment can vary between physicians as clinical guidelines (concerning detection, diagnosis, and treatment) only provide recommendations. Interventions for these patients can include medication in combination with conversational therapy (psychoeducation) and support (high accessibility and continuity, early next appointment, guided self-help) [38]. Treatment can also include contact with an occupational therapist, rehabilitation coordinator, or physiotherapist and group sessions targeting specific symptoms or problems depending on available services at each primary health care centre.

### Intervention group

The intervention, consisting of PCC delivered via an interactive digital platform (“My Health”), as well as phone support, was provided in addition to TAU. The team of HCPs (n = 5) conducting the intervention represented different disciplines (e.g. nursing, physiotherapy and occupational therapy). All received a half-day education regarding CMDs and the philosophical standpoints of PCC led by specialists for each area. They also had access to a forum where they, together with specialists in PCC, could raise questions and share experiences of practising PCC. The first phone conversation was jointly scheduled between HCPs and patients in the intervention group and a web link was sent to patients for access to the digital platform. Additional follow-ups by phone were agreed and scheduled as required. The intervention aimed to apply the ethics of PCC in practice and was designed to facilitate a partnership between patients, professionals, and, if desired, the patients’ extended social network.

The telephone support consisted of an HCP listening attentively to the patient’s narratives about their daily life and current situation. The HCPs encouraged narration and established a partnership using communication skills such as asking open-ended questions, sharing reflections and summarizing. Based on the patient’s narrative a health plan was co-created, which captured the patient’s experiences of their situation, capabilities, resources, needs and goals. The health plan was uploaded to the digital platform and served as a guide to be used together by the patient and HCPs in further phone conversations and communication via the platform.

The platform contained functions to facilitate patients’ self-management. Thus, the patients were invited to use a diary function for free-text entries and to rate their daily symptoms, visualized as trend graphs that allowed them to follow symptoms and their recovery process over time. Patients and HCPs communicated via the platform in a chat-like forum, and patients were able to invite and give customized access to the platform to any person they wanted, such as family, friends, and workplace representatives. They also had access to links to relevant websites containing information about CMDs. A participatory design was used in which the researchers organized workshops with patient representatives, system developers and HCPs to discuss and develop the PCC eHealth intervention [39].

### Data collection and outcome measures

Self-reported questionnaire data were gathered by regular mail at baseline and after 3 and 6 months. The level of burnout was assessed using the SMBQ [40], which can be used as screening tool and to assess severity of symptoms in clinical settings with the scale showing good, discriminated validity in separating between clinical cases of ED and healthy individuals [20]. The SMBQ originally contained 22 items with four subscales: physical fatigue, cognitive weariness, tension, and listlessness. A Swedish revised version containing 18 items, excluding tension, was used in the present study [20]. The items in the form of statements are answered on a 7-point scale ranging from 1 (“almost never”) to 7 (“almost always”). Instead of calculating the mean score for the 18 items, a transformed metric score was calculated as recommended. The possible score ranges from 18 to 126 (corresponding mean values are 1 to 7), with higher values indicating a higher degree of burnout. A value of 79 is considered as a cut-off for clinical burnout and corresponds to the mean score 4.4. The complete table with equating values metric to mean values can be found in Lundgren-Nilsson et al. [20].

### Statistical analysis

Descriptive statistics are given in percentages and numbers for categorical variables and means and standard deviation (SD) for continuous variables. Longitudinal associations between the two groups (the intervention and control group) and SMBQ scores were analysed using linear mixed models with random intercept in SPSS version 25 (IBM, Armonk, NY, USA). Age and gender were tested as possible confounders and the inclusion criterion was p < 0.25. Time was included as continuous variable (0, 3 and 6 months from inclusion in the study). Interactions between the groups and time were tested to evaluate whether the development over time was different in the two groups. Parameter estimates along with 95% confidence intervals (CIs) are presented as a measure of association.

Post hoc analysis was done to understand the nature of the interaction effect. Independent-sample *t*-test between the two groups was performed to evaluate the magnitude of change expressed as the raw score difference between baseline and 3 and 6 months, as well as the change between 3 and 6 months.

## Results

The study population consisted mostly of women (84%, n = 175) with a mean age of 42 (SD 11.45) years. In total, 134 (64%) of the participants had stress disorders, 44 (21%) depression and 31 (15%) anxiety disorders. The intervention and control groups’ baseline characteristics (e.g. age, civil status, education level, illness history, current medication) were comparable (Table 1). At baseline, the mean SMBQ scores did not differ significantly between groups. The response rate on the self-reported questionnaire was relatively high and similar in both groups, 72.0% in the control group vs. 74.5% in the intervention group at three months follow-up, and 82.2% in the control group vs. 74.5% in the intervention group at the six months follow-up.

**Table 1:** Baseline characteristics

	Control group n = 107	Intervention group n = 102
Age, yrs, mean (SD)	42.2 (11.7)	42.3 (11.2)
Gender, n (%)		
Female	93 (87.7) ^a^	82 (80.4)
Shirom-Melamed Burnout Questionnaire (SMBQ) score, mean (SD)	86.0 (8.1)	87.7 (9.9)
Civil status, n (%)		
Married/living with a partner Living alone	77 (72.0) 30 (28.0)	62 (60.8) 40 (39.2)
Country of birth, n (%)		
Sweden Other	91 (85.0) 16 (15.0)	89 (87.3) 13 (12.7)
Diagnosis (with ICD codes), n (%)		
Stress (F43) Depression (F32 and F33) Anxiety (F41)	69 (64.5) 23 (21.5) 15 (14.0)	65 (63.7) 21 (20.6) 16 (15.7)
Education level^b^, n (%)		
Compulsory schooling Secondary school Vocational college University	7 (6.6) 16 (15.1) 20 (18.9) 63 (59.4)	6 (5.9) 21 (20.8) 15 (14.9) 59 (58.4)
Current sick leave, n (%)		
0 25 50 75 100	4 (3.7) 3 (2.8) 21 (19.6) 3 (2.8) 76 (71.0)	2 (2.0) 5 (4.9) 30 (29.4) 5 (4.9) 60 (58.8)
Illness history, n (%)		
Previous stress Previous anxiety Previous depression Previous sleep disorder	29 (27.4) 33 (31.1) 28 (26.4) 15 (14.0)	34 (33.3) ^a^ 29 (28.4) ^a^ 30 (29.4) ^a^ 17 (16.7)
Current medication, n (%)		
Antidepressants Sedatives Sleep medication	54 (50.5) 49 (46.2) 26 (24.5)	43 (42.2) 37 (36.3) ^a^ 25 (24.5) ^a^

^a^ One value missing

^b^ Two value missing

SD = standard deviation

ICD = International Statistical Classification of Diseases and Related Health Problems

In the intervention group, 99 (97.1%) used the phone support at least once during the 6-month study period (mean number of conversations = 4.05, SD 1.84), with an average of 32 min per conversation. 74 (72.5%) used the function of self-ratings (number of ratings = 1,415, mean 19.12, SD 27.23). Sixty per cent (n = 245) of the phone conversations and 82% (n = 1155) of the self-ratings were made during the first 3 months.

**Fig. 2:**
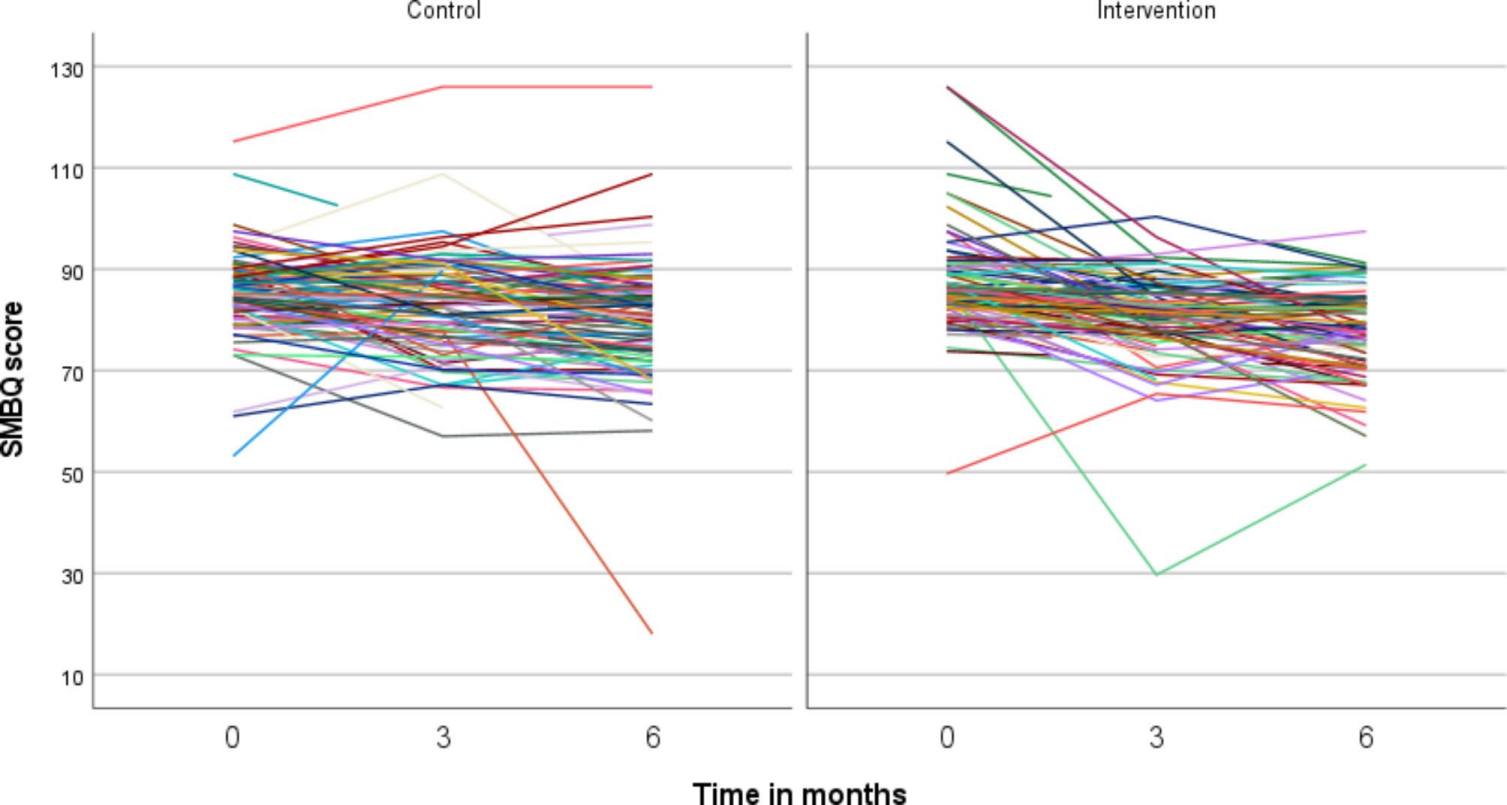
Shirom-Melamed Burnout Questionnaire (SMBQ) scores at each time point for each patient in the intervention and control group

**Fig. 3:**
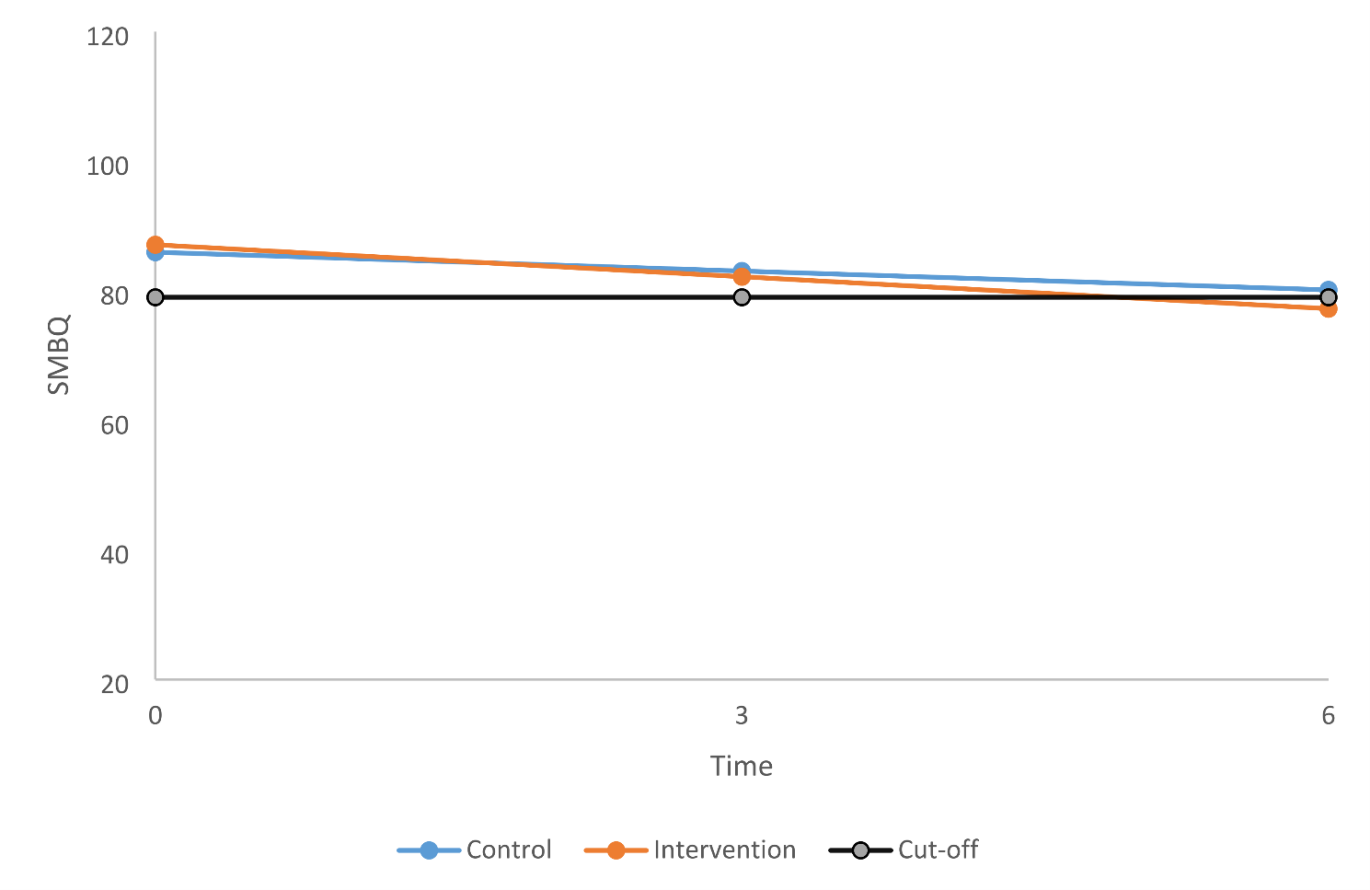
Estimated mean score on the Shirom-Melamed Burnout Questionnaire (SMBQ) for the intervention and control groups at each time point

As shown in Fig. 2, which presents SMBQ scores for each patient at each time point, the individual variation was large but the overall trend for both groups seemed to be a decrease in SMBQ scores over time. This observation was also confirmed by our analyses presented in Table 2. Time was significantly associated with SMBQ scores and the average decrease over time was − 1.64, with 95% CIs ranging from − 1.29 to -2.00 points on the SMBQ scale. Age and gender were evaluated as possible confounders but were not significant and were therefore not included in the final model. As seen in Table 2, there was no significant difference in SMBQ scores between the groups; however, the interaction between time and groups was significant, which means that the change over time differed between the groups. These results are visualized in Fig. 3, which shows estimated means for each group and time point as well as the value for the clinical cut-off (SMBQ score of 79). The intervention group started at a slightly higher average level compared with the control group (estimated mean 87.1 and 86.0, respectively), which shifted at 3 months (82.2 and 83.9, respectively). At 6 months the estimated mean SMBQ score was below the clinical cut-off for burnout in the intervention group (77.2) while the control group remained just above the threshold (80.1) (Fig. 3).

**Table 2:** Estimates of the longitudinal association between the intervention and control groups and their Shirom-Melamed Burnout Questionnaire (SMBQ) scores. CI = confidence interval

	Estimate	95% CI	Type III p-value
Intercept	87.11	85.24; 88.97	< 0.0005
Intervention group	-1.15	-3.75; 1.45	0.385
Control group	0		
Time	-1.64	-2.00; -1.29	< 0.0005
Intervention*time	0.68	0.19; 1.16	0.007

Post hoc analyses were performed to explore the nature of the interaction effect, which showed that both groups had decreased their SMBQ scores between baseline and the 3- and 6-month follow-up, but that the SMBQ scores for the intervention group scores decreased more than for the control group. At the 3-month follow-up the average decrease in the intervention group was − 7.7 points (SD 10.5) compared with − 2.4 (SD 8.5) in the control group, a statistically significant difference (mean difference 5.3, p = 0.001). Between baseline and the 6-month follow-up the corresponding figures were − 9.8 (SD 11.6) compared with − 5.8 (SD 10.2) (mean difference 4.0, p = 0.020). There was no significant difference in change between the groups between the 3- and 6-month follow-up (Table 3).

**Table 3:** Average change in Shirom-Melamed Burnout Questionnaire (SMBQ) scores within each group, and differences in change between groups and time points. CI = confidence interval; SD = standard deviation

	Population	Mean change (SD) within each group	Mean differences between the groups (95% CI)	P-value
Baseline – 3 months	Control (n = 77)	-2.4 (8.53)	5.3 (2.27; 8.39)	0.001
	Intervention (n = 76)	-7.7 (10.54)		
3–6 months	Control (n = 74)	-3.3 (9.63)	− .74 (-3.59; 2.11)	0.608
	Intervention (n = 70)	-2.5 (7.44)		
Baseline – 6 months	Control (n = 88)	-5.8 (10.17)	3.98 (0.63; 7.33)	0.020
	Intervention (n = 76)	-9.8 (11.55)		

## Discussion

The present study in patients on sick leave for CMDs aimed to compare levels of self-reported symptoms of burnout between a control group receiving TAU and an intervention group receiving a person-centred eHealth intervention in addition to TAU. Our findings showed changes in SMBQ scores over time in both groups. There was no significant difference in SMBQ scores between the groups. However, there was a difference in change over time between the groups. The SMBQ scores for the intervention group decreased significantly more compared to the control group between 0 and 3 months and between 0 and 6 months. No differences in change between the two groups were seen between the 3- and the 6-month follow-ups. Overall, the effect of the intervention should be considered small given that both groups had comparable levels of SMBQ throughout the study period. The cut-off for clinical burnout for the SMBQ is 79 [20], and the intervention and control groups started with an estimated mean score of 87.1 and 86.0, respectively. At 6 months, the estimated mean SMBQ score was below the clinical cut-off for burnout in the intervention group (77.2) while the control group remained just above the threshold (80.1), indicating a slight decrease in SMBQ levels but an overall high level of burnout in both groups during the 6-month study period.

Previous research has shown that Internet-based interventions are an equivalent alternative to face-to-face treatments [30, 41]. However, studies have also shown that treatments and interventions for CMDs have limited effects on symptom relief [32, 42]. It is worth noting that the study population reported a high average level of burnout during the six-month study period (both for the controls and for the intervention group) even if half of the patients at the 3-month follow-up, and 70% at the 6-month follow-up, no longer reported ongoing sick leave [43]. This aligns with previous research showing that not being on sick leave does not necessarily mirror a patient’s symptom relief [4, 44]. The slight effect observed at the 3-month follow-up may be related to patients making more use of the PCC eHealth intervention during the first 3 months (during which 60% of the phone conversations and 77% of the self-ratings on the platform were made). As most of the patients no longer were on sick leave between 3 and 6 months, they may also have considered their condition as improved and, therefore, they may have considered themselves in less need of using the PCC eHealth intervention.

Even though our results showed that the intervention had a limited effect, we cannot rule out that it relieved the symptom burden for some of the participants during the first 3 months. One possible advantage of the eHealth support may have been that the intervention was performed early in the sick leave period. Interventions in the early stages of sick leave have been shown to prevent both deteriorating conditions and long-term sick leave with risks of relapse [45]. The Swedish National Board of Health and Welfare recommends periods of sick leave of 2 weeks for adjustment disorders, 2 months for depression and 6 months to 1 year, or more, for ED [46]. All these conditions include symptoms of stress and burnout [15], and even if conditions such as adjustment disorders do not necessarily require a health care intervention, they can decrease quality of life and pose a risk of developing more severe mental disorders [26].

The fact that the intervention was administered from a distance may have increased accessibility for patients at risk of social isolation and patients occasionally experiencing resistance to a scheduled time in primary care [47] as it allowed patients to access the platform at any time and in any place. Previous studies have shown that interventions at a distance are a feasible option for face-to-face treatment in patients with CMDs [30, 31]. Moreover, distance interventions have been reported to improve self-management by increasing accessibility and direct patient involvement [32]. The remote setting of the intervention also had its advantages in view of the COVID-19 pandemic as the participants were able to receive support regardless of social restrictions. The intervention was performed in a research setting, separately and in addition to TAU, and there was no interaction between the team of HCPs conducting the intervention and other HCPs in the context of TAU. Most participants reported high educational level which is possibly somewhat unexpected as previous studies have shown a relationship between lower socioeconomic status and higher sickness absence due to CMDs [48]. However, a recent study reported that sick leave due to CMDs was more common among people with higher educational levels than those with lower educational levels. One plausible explanation is that work situation requiring higher education are more demanding with regard to psychosocial work load, which could likely lead to stress-related mental health problems [49].

Van Dam suggests providing tailored interventions and treatment as patients require different treatment approaches and mental illness prevention strategies [15]. Generally, the evidence for treatment of stress-related disorders (e.g., adjustment disorder and clinical burnout or exhaustion disorder) is scarce [50, 51]. Regarding stress disorders and ED, Ellbin and co-workers [45] found that patients’ personal patterns and values contribute to both the onset and the maintenance of ED. Moreover, patients are entrenched in relational, family-related and work-related systems, making individualized treatment necessary [45].

In summary, our results show that the PCC eHealth intervention had a limited effect, but we cannot exclude that it relieved the symptom burden for some participants during the first 3 months and may be a feasible option for some patients. In health care encounters, professionals need to listen carefully to patients’ narratives and acknowledge their unique needs and resources in order to tailor the support based on each patient’s situation [29, 30].

### Study limitations

Because this study is a secondary outcome analysis and was not initially used to detect differences in symptoms of burnout, a power calculation was not performed. The response rate on the self-reported questionnaire was relatively high (75%). However, there remains a risk of non-response bias. Another area for improvement is that the SMBQ might not precisely reveal the clinical picture of the different diagnoses included in CMDs, which may have impacted the results. Being involved in a research study might also have affected participants’ motivation to engage in TAU.

## Conclusion

Our study showed that both groups reported comparable, and overall high, levels of symptoms of burnout during the study period. The person-centred eHealth intervention for patients on sick leave for CMDs had a slight effect in reducing symptoms of burnout. Further research is needed to evaluate the intervention’s long-term effects and determine the clinical value of these findings.

## Data Availability

As dictated by the ethical body that approved the study and the promise to participants in their informed consent, the raw study data cannot be shared publicly as the data contain potentially identifying or sensitive patient information. The datasets used and/or analyzed during the current study are available from the corresponding author on reasonable request.
